# PET imaging studies show enhanced expression of mGluR5 and inflammatory response during progressive degeneration in ALS mouse model expressing SOD1-G93A gene

**DOI:** 10.1186/s12974-015-0439-9

**Published:** 2015-11-24

**Authors:** Anna-Liisa Brownell, Darshini Kuruppu, Kun-Eek Kil, Kimmo Jokivarsi, Pekka Poutiainen, Aijun Zhu, Michelle Maxwell

**Affiliations:** Athinoula A Martinos Biomedical Imaging Center, Imaging, Massachusetts General Hospital, 149 13th Street, Charlestown, MA USA; Department of Surgery, Massachusetts General Hospital, Charlestown, Massachusett USA; Department of Neurology, Massachusetts General Hospital, Charlestown, Massachusett USA

**Keywords:** ALS, Glutamate, mGluR5, Inflammation, [^18^F]FPEB, [^11^C]PBR28

## Abstract

**Background:**

Amyotrophic lateral sclerosis (ALS) is a progressive neurodegenerative motor neuron disorder. Genetic studies have linked mutation of the gene SOD1 to ALS pathology as well as several other pathological processes including modulation of glutamatergic function and inflammatory processes. Since therapeutic approaches for ALS are focused on glutamatergic function, we investigated modulation of glutamate transport based on its receptor function as well as excitotoxicity-induced inflammatory response.

**Methods:**

In vivo positron emission tomography (PET) imaging studies of metabotropic glutamate receptor subtype 5 (mGluR5) using [^18^F]FPEB ([^18^F]3-fluoro-5-(2-pyridylethynyl)benzonitrile) and inflammatory response using [^11^C]PBR28 (peripheral benzodiazepine receptor ligand 28) were done in an early and a late phase of neurodegeneration in four ALS mice expressing SOD1-G93A gene and four control base mice (C57/BL6). Accumulation of [^18^F]FPEB and [^11^C]PBR28 were quantitated in several brain areas and spinal cord to determine degeneration-induced modulation. The studies were completed with immunohistochemical analyses of mGluR5 and inflammatory response.

**Results:**

These studies showed enhanced binding potential of [^18^F]FPEB in several brain areas including striatum, hippocampus, and frontal cortex. In the whole brain, the binding potential increased 49 ± 9 % from base mice to ALS-type mice and further enhanced 23 ± 4 % during disease progression. Also, in the spinal cord 6–22 %, enhanced accumulation of [^18^F]FPEB was observed during progression of the disease. The accumulation of [^11^C]PBR28 increased by 110 ± 33 % in the whole brain during progression of the disease indicating significant inflammatory process. [^11^C]PBR28 accumulation enhanced 89–264 % in the spinal cord and 204 % in the lungs. The end point immunohistochemical analyses verified the enhanced mGluR5 expression and inflammation.

**Conclusions:**

These results confirm the role of glutamate and inflammation in ALS-type pathology. These data also support the hypothesis that excessive glutamate may contribute to inflammation in the chronic neurodegenerative processes in ALS.

## Background

Amyotrophic lateral sclerosis (ALS) is a devastating neuromuscular disorder without known cause or curative treatment. The pathological processes linked to ALS are formation of intraneuronal aggregates of neurofilament [1]; microglia mediated neurotoxicity as a result of enhanced inflammatory response [2–5]; glutamatergic neurotoxicity as a result of deficit in glutamate transporter or receptor function [6–11]; and mitochondrial dysfunction as a result of failure in oxidative metabolism [12, 13] and superoxide dismutase-1 (SOD1) mediated neurotoxicity, which is associated to gene mutation and found in familiar form of ALS [14, 15]. Altogether, ALS is a multimodal disorder and it is not yet known what mechanism ignites the fast progression of the disease.

For the treatment of ALS, the Food and Drug Administration has approved one drug, riluzole, which inhibits glutamate release. Another drug, ceftriaxole, which belongs to penicillin family, increases glutamate transport and is used in clinical trials [16, 17]. Thus, glutamate-related research is an essential part for development of diagnosis and treatment of ALS. It has been shown that ALS patients have enhanced glutamate levels in serum and spinal cord. Extracellular glutamate can induce neurotoxicity by either increasing neuronal sodium and chloride influx during depolarization or increasing calcium influx. Increased calcium influx may lead to activation of several calcium-dependent enzymes with end point damage in DNA and mitochondrial dysfunction [1, 7]. It is noticeable that neurotransmitter glutamate does not cross blood-brain barrier but its function can be investigated based on its receptors.

Metabotropic glutamate receptors (mGluRs) have various physiological functions in the central nervous system by affecting on several intracellular signal transduction mechanisms through G-protein [18, 19]. A large amount of pharmacological agents acting on metabotropic glutamate receptors have appeared in literature. Starting from 1996 [20], several non-competitive negative, positive, and neutral allosteric modulators have been developed as mGluR ligands [21, 22]. These ligands modulate mGlu receptor activity by binding to allosteric binding sites that are located in the seven strand transmembrane domains. The allosteric binding site is structurally distinct from the classical agonist or antagonist orthosteric binding site [23].

The glial cells that usually support and nourish neighboring neurons in the nervous system can become overactive in certain conditions. If glia becomes too activated, it can damage the tissue. When glutamate is taken up into glial cell by excitatory amino acid transporters, it is not reused directly but converted to glutamine and stored in vesicles. Subsequently, these vesicles are released from glial cells, and glutamine is transported back into presynaptic neuron and converted back to glutamate [23]. Excitotoxicity, which is mediated by the excessive activation of glutamate receptors, has been implicated in the pathogenesis of ALS. Aronica et al. [24] demonstrated with immunocytochemical analyses that mGluR5 expression was high in neuronal cell throughout the human spinal cord with the highest expression in ventral horn neurons. Regional differences in immunoreactivity were observed in ALS compared to control studies. Especially, mGluR5 expression was increased in reactive glial cell in both gray (ventral horn) and white matter of ALS spinal cord [24]. Upregulation of mGluRs in reactive astrocytes may represent a critical mechanism for modulation of glial function and changes in glial-neuronal communication in chronic neurodegeneration [24]. Activated microglial cells can play a major role in the neuronal loss after a lesion or an insult, such as neurotoxin exposure [25]. There is increasing evidence that inflammation accompanies the death of motor neurons in ALS [26].

We have developed several allosteric modulators to image metabotropic glutamate subtype receptors 5 (mGluR5s) [27–29] and characterized their in vivo dynamic biodistribution and functional binding properties in different experimental animal models including ALS mouse model using in vivo microPET imaging studies [30–32]. Here, we report preliminary positron emission tomography (PET)/computerized tomography (CT) imaging studies of modulation of mGluR5 expression and CNS inflammation during progressive degeneration in ALS mouse model carrying SOD1-G93A gene.

## Methods

### In vivo PET imaging studies

All animal studies were approved by the subcommittee on research animals of the Massachusetts General Hospital and Harvard Medical School and carried out by the guidance of the National Institute of Health Guide for the Care and Use of Laboratory Animals.

In vivo PET imaging studies were conducted in four base mice (C57/BL6) and four transgenic mouse model of ALS expressing human gene, SOD1-G93A (superoxide dismutase 1-glycine 93S mutation), to investigate synaptic glutamatergic function and inflammatory response during progression of the disease. The used PET imaging ligand for mGluR5, [^18^F]FPEB ([^18^F]3-fluoro-5-(2-pyridylethynyl)benzonitrile) [27] is developed by our team and it is presently in a routine production. Imaging of inflammatory response was done using [^11^C]PBR28 (peripheral benzodiazepine receptor ligand 28), an imaging ligand for translocator protein 18kDA (TSPO) which is a biomarker for inflammation and activated microglia.

For imaging studies, animals were anesthetized with isoflurane/nitrous oxide/oxygen (1–1.5 % isoflurane at 1 l/min flow) and placed on the imaging table. Catheterization of tail vein was done for the administration of the radiolabeled ligands. The animal head was secured in a plexiglass custom-made head holder equipped with ear and mouth bars designed to ensure reproducible head positioning. Level of anesthesia and vital signs was monitored throughout the imaging procedures using the physiological monitoring system included to the imaging device (Triumph II, Trifoil Imaging, Inc).

After the animal was adjusted into the imaging position, a computerized tomography (CT) imaging was done before administration of the radioactivity to obtain high-resolution anatomical information and data for attenuation correction. Radiolabeled ligand, [^18^F]FPEB or [^11^C]PBR28, (0.1–0.2 mCi, specific activity of 1900 mCi/μmol for [^18^F]FPEB or 0.2–0.3 mCi of [^11^C]PBR28) was injected into the tail vein. The radioactivity was diluted into the saline in portion 1 to 10 before the injection to decrease the ethanol concentration that was 10 % after the production of the ligand. Dynamic volumetric imaging data were acquired for 60 min after the administration of the radiolabeled ligand. Imaging data were corrected for uniformity, sensitivity, scatter, attenuation, decay, and acquisition time. PET images were reconstructed using a volumetric maximum likelihood estimation method (three-dimensional maximum likelihood estimation method) with 30 iterations (the software provided by the manufacturer of the scanner). The regions of interest including striatum, frontal cortex, hippocampus, cerebellum, whole brain area, and several areas in spinal cord were drawn on all coronal and axial levels, visualized in the fused PET-CT images. Activity per unit volume, percent activity of injected dose, and the ligand concentration were calculated.

Studies of mGluR5 expression with [^18^F]FPEB were done at first and followed by studies of inflammation with [^11^C]PBR 2 days later. To follow the progressive degeneration, the PET imaging studies were repeated 2 weeks later. The experimental studies of neurodegeneration were completed with immunohistological studies (IHS) including expression of mGluR5 and inflammation with IBA1.

Kinetic analysis of [^18^F]FPEB data obtained from brain was done using the Pmod software package (Pmod Technologies, Zurich, Switzerland) to determine regional values for binding potential [29, 33, 34]. Since rodent cerebellum does not express mGlu5 receptors or the expression is minimal, the input function can be processed from the data obtained from cerebellum in calculating regional maps for binding potential [27, 29]. Concerning [^11^C]PBR28, cerebellum cannot be used as a reference area, since inflammation is not brain area specific. For that reason, the accumulation of [^11^C]PBR28 was expressed as percent of the injected dose per cm^3^ (%id/cm^3^).

Scoring of the progressive degeneration was determined according to the following criteria: if the hind legs were fully extended away from lateral midline when mouse was suspended by its tail and the mouse could hold this for 2 s when suspended 2–3 times the score was 1 and if two toes curled under at least twice during walking of 12 in. or any part of foot was dragging along cage bottom/table the score was 3. The scoring model was modified from the behavioral model of Knippenberg et al. [35] in ALS mice.

### Statistical analyses

Statistical analyses between the control and ALS mice as well as during progressive degeneration were done using Student’s *t* test.

### Immunohistochemical studies

The mice were anesthetized with sodium pentobarbital (60 mg/ml, i.p. (0.1 ml/100 g)) and perfused transcardially with heparinized saline followed by 4 % paraformaldehyde (PFA) in 0.1 M phosphate-buffered saline, pH 7.4. The brain and spinal cord were removed and fixed in 4 % formalin for 5 days. They were processed for immunohistochemical staining by dehydrating through a series of increasing ethanol concentrations (50, 70, to 100 %) followed by immersing in xylaxine to enable paraffin embedding. Paraffin blocks with the brain and spinal cord were prepared and sectioned using a microtome at 8 μm. The sections were air dried overnight before immunostaining. Each slide with three serial sections of the brain and spinal cord was immersed in xylaxine to dewax the sections. The tissues were rehydrated through increasing ethanol concentrations and finally in distilled water before antigen retrieval in citrate buffer for 5 min. The sections were washed and outlined with a PAP pen before blocking for endogenous peroxidase for 1 h. Non-specific antibody was blocked with goat serum for 1 h. One serial section was incubated with antibody for mGluR5 (Abcam) applied at 1:200 dilution, while the adjacent section was incubated with IBA1 antibody (Abcam) at 1:100 dilution. The sections were incubated with the respective antibodies overnight at 4 °C. The negative control did not have the primary antibody. On the following day, the sections were washed before applying biotinylated goat anti-rabbit secondary antibody for 1 h. After that, the slides were incubated in streptavidin-HRP for 30 min. The antibody was detected with the chromogen 3,3′-diaminobenzidine (DAB). The secondary antibodies with developing reagents were purchased from Millipore. The nuclei were stained with hematoxylin. The sections were washed after each step before subsequent application. The stained mGluR5 positive cells and IBA1 positive microglial cells were viewed under a light microscope.

## Results

**Fig. 1:**
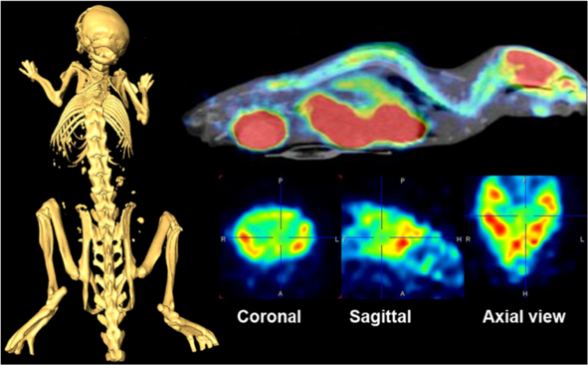
PET, CT, and fused PET/CT images illustrate anatomical distribution of [^18^F]FPEB, a mGluR5 imaging ligand in an ALS mouse. Separate CT (*left*), PET (*right bottom*), and fused PET/CT (*right above*) images of an ALS mouse at stage 3. In the fused image, the sagittal slice (thickness 0.6 mm) shows enhanced accumulation of [^18^F]FPEB in brain, spinal cord, abdominal area, and bladder 60 min after administration of radioligand (120 μCi iv). Coronal, sagittal, and axial views of the brain in the PET study show high accumulation in cortical areas. Crosshair in the images illustrates the location of each slice. In these mice, we have observed progressively enhanced mGluR5 expression when the general health status declined. The CT image demonstrates the level of spatial detection that can be achieved with the present imaging technology. We can accurately detect and quantitate modulation of receptor binding and inflammatory response in small areas in spinal cord and brain

**Fig. 2:**
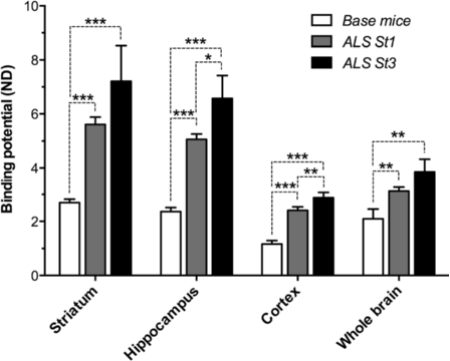
Quantitative distribution of [^18^F]FPEB in different brain areas in the base mouse and ALS mouse at the stages 1 and 3. Comparison of the binding of [^18^F]FPEB in the striatum, hippocampus, cortex, and whole brain in base mice and ALS mice at the stages 1 and 3. It can be seen that mGluR5 expression is highly increased in ALS mice carrying SOD1-G93A gene compared to base mice and it is further increased when the disease progressed. Of the individual brain areas, the hippocampus has the highest increase between the base mice and ALS mice and further the highest increase between stages 1 and 3 making the hippocampus area the most sensitive brain area for glutamatergic modulation in ALS-type degeneration. Values for binding potential (ND) were calculated based on reference tissue model using cerebellar data as an input function [27, 33, 34]. Mean values for binding potential were calculated from four base mice and four ALS mice. Statistical analysis was done with student’s *t* test, and significances are marked to the figure (*p < 0.05 = , **p < 0.01 = , and ***p < 0.001 = )

**Fig. 3:**
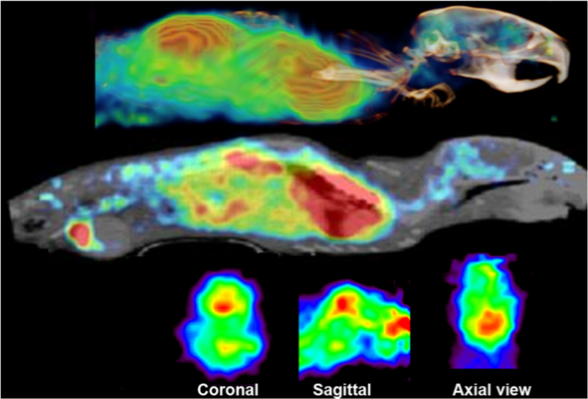
PET and fused PET/CT images of the distribution of [^11^C]PBR28 accumulation in ALS mouse at the stage 3. Studies of inflammatory response using [^11^C]PBR28 (240 μCi i.v.) were conducted in the same mouse as above (Fig. 1) 2 days after the study of mGluR5 expression. High accumulation of [^11^C]PBR28 was seen in lungs, hindbrain, brain stem, and in different parts of spinal cord, indicating activated microglia and expression of translocator protein (TSPO). In the mice, we observed fivefold higher enhancement of inflammatory response in brain stem and cervical canal in progression of the disease than in modulation of mGluR5 expression in the same areas

**Fig. 4:**
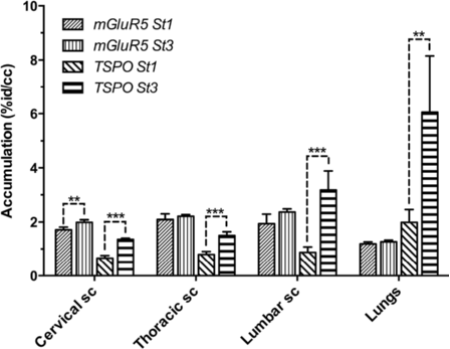
Quantitative analysis of modulation of mGluR5 expression and inflammatory response in the spinal cord and lungs during progression of the disease. Comparison of mGluR5 expression and inflammatory response during progression of ALS-type degeneration in different parts of spinal cord and lungs shows enhancement in the accumulation of [^18^F]FPEB but significantly higher enhancement of [^11^C]PBR28 accumulation in the lumbar cord and lungs. The selected region of interest was the same for both studies. Quantitative values were calculated as percent of the injected activity in the tissue volume (cm^3^) since no reference tissue method can be used for inflammatory processes as well as no brain-related input function for peripheral mGluR5 expression. Mean values for accumulation were calculated from four base mice and four ALS-mice. Statistical analyses were done with student’s *t* test, and significances are marked to the figure (*p < 0.05 = , **p < 0.01 = , and ***p < 0.001 = )

**Fig. 5:**
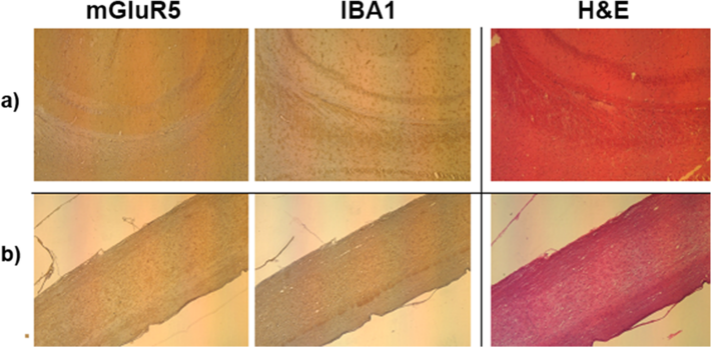
Immunohistochemical studies of expression of mGluR5 and IBA1 in ALS brains and spinal cord. mGluR5 and IBA1 expression in the brain (**a**) and spinal cord (**b**) of ALS mice showed the active inflammation occurring in these regions following neurotransmitter release. mGluR5 was highly expressed in the entire brain, primarily in the gray matter. In the caudal diencephalon, mGluR5 expression was found in the striatum and hippocampus regions. Microglia which was stained by IBA1 was expressed at sites consisting of capillaries and neurons in these regions. In some areas, the microglial cell bodies were enlarged, while in some areas, the microglia were elongated. Both these morphological forms of microglia were indicative of inflammation. mGluR5 was expressed throughout the spinal cord and IBA1 was expressed in the gray matter as an indication of inflammatory processes

The binding potential (BP_ND_) of [^18^F]FPEB in the whole brain of the base mice was 2.09 ± 0.36; in ALS mice at stage 1, it was 3.13 ± 0.15 and at stage 3 3.85 ± 0.47 indicating 49 ± 9 % increase from the base mice to ALS-type mice and further enhancement of 23 ± 4 % during disease progression. In the individual brain areas, the highest increase was in the hippocampus being 115 ± 15 % from base mice to ALS mice and further 30 ± 5 % during degeneration. The corresponding values for the binding potential in the striatum were 2.69 ± 0.13 in the base mice, 5.61 ± 0.27 in ALS mice at stage 1, and 7.22 ± 1.31 at stage 3 indicating 108 ± 11 % increase from the base mice to the ALS mice and 29 ± 5 % increase during progression of the disease. Correspondingly, the binding potential in the cortex of the base mice was 1.17 ± 0.12, in the ALS mice at stage 1 2.41 ± 0.13, and 2.88 ± 0.19 at stage 3 indicating 105 ± 11 % increase from the base mice and further increase of 19.5 ± 3 % during disease progression (Figs. 1 and 2). The accumulation of [^11^C]PBR28 was increased by 110 ± 33 % in the whole brain during progression of the disease indicating significant inflammatory process (Fig. 3). The accumulation of [^18^F]FPEB was enhanced 6 % in thoracic spinal cord and 22 % in lumbar cord while [^11^C]PBR28 accumulation was enhanced 89 and 264 %, respectively, in the same areas during progression of the disease. Also, in the lungs, [^11^C]PBR28 accumulation was increased by 204 % indicating excessive inflammatory process. [^18^F]FPEB accumulation was enhanced only 6 % in the lungs (Fig. 4). The immunohistological studies verified enhanced mGluR5 expression and inflammatory response in the brain and spinal cord (Fig. 5).

## Discussion

Compared to the base mice, the mGluR5 expression was significantly enhanced in the brain of ALS mouse model especially in the hippocampus, striatum, and cortex as well as in the averaged whole brain enhancing significantly also during progression of the disease in the hippocampus and cortex. Significant enhancement of mGluR5 expression was observed also in the cervical spinal cord during the disease progression. However, [^11^C]PBR28, which is a marker for TSPO and microglial activation, showed the most significant enhancement in the brain and spinal cord during progression of the disease. The enhancement in the whole brain was 110+/33 %, five times more than averaged enhancement of mGluR5 expression. In the spinal cord, the enhancement was from 89 to 264 % and in the lungs 204 %. The difference between enhancements of mGluR5 expression and inflammatory response might be caused by the excessive glutamate-induced excitotoxicity in the synapses which may contribute to enhanced inflammatory processes in CNS.

Concerning the imaging ligand [^11^C]PBR28, the accumulation in the lungs might be affected by non-specific binding, which has been reported for [^11^C]PBR28 in the lungs [36]. In addition, TSPO has polymorphism that effects binding potential in vitro and in vivo in humans [37]. In this experiment, we followed the same animals with the same imaging procedures so even if the non-specific binding or polymorphism effects on absolute values, their effect on the difference between the values is minimal.

Since inflammation can occur in any brain area, reference tissue model cannot be used to analyze [^11^C]PBR28 data. These data are presented as percent accumulation of the injected radioactivity per tissue volume. The selected regions of the interest are the same as for the studies with [^18^F]FPEB. During the disease progression from stage 1 to 3, inflammatory response increased five times more than mGluR5 expression in the whole brain providing strong evidence that inflammation can be dependent on enhanced glutamatergic function. Since mGlu5 receptors have primary postsynaptic location, the enhanced binding potential values of mGluR5 may indicate enhanced glutamate content in the synapse.

Cerebellum data cannot be used as a reference tissue in the data analyses of [^18^F]FPEB in the spinal cord since the transportation of the radioligand is not the same as inside the brain, so expression was determined as a percent of the injected dose in cubic centimeter.

Resolution of the microPET imager is 0.9 mm, which will create errors in absolute values in quantification of data in small brain areas because of the partial volume effect. To minimize this issue, we have conducted longitudinal imaging studies in the same animals to evaluate functional changes and have used the same size for region of interest in consecutive studies. To be noted that the resolution of the CT is 50 μm enabling us to localize very tiny areas even in the spinal cord but when we record the radioactivity in that tiny area, the absolute value will be underestimated.

The obtained values for excessive inflammation support the fast decline of the health status of these mice, and the immunohistological studies verify the end point data of enhanced mGluR5 expression and inflammation in the brain and spinal cord.

Using the powerful imaging technology, we have conducted imaging studies of mGluR5 expression and inflammatory response in SOD1 G93 mice and found enhanced mGluR5 expression in several brain areas and spinal cord with enhancement in 2 weeks follow-up period (Figs. 1 and 2). We found also significantly enhanced accumulation of [^11^C]PBR28, marker for activated microglia, in hind brain, areas in spinal cord, and the highest accumulation in the lungs (Figs. 3 and 4). The enhanced expression of mGluR5 in the brain of ALS mice support the regional inflammation observed in ALS patients using [^11^C]PBR28 as an imaging ligand [38]. The regional areas of the brain in these human studies were determined by the simultaneous anatomical MR imaging. These studies also showed that [^11^C]PBR28 binding was enhanced in all brain areas of ALS patients compared to age-matched controls.

The studies on ALS pathology based on glutamatergic function and inflammatory response using the highly innovative in vivo imaging technology recently developed by our team [27, 30] show significant local pathophysiological changes that can be targeted with therapeutic approaches. The results of these exploratory experiments can be immediately extended to ALS patients to enhance early diagnosis and follow therapeutic response and even to design more effective therapeutic approaches. Imaging ligands of mGluR5 ([^18^F]FPEB) and inflammatory response ([^11^C]PBR 28) are already in human use, so the technical aspects for transition are immediate. It is notable that there have been several failed clinical trials in ALS. One contributing matter for this might be that previously, there has been limitation to spatially and temporally record involved pathophysiological mechanisms.

## Conclusions

With in vivo PET imaging studies, we have shown that SOD1-G93A gene will significantly enhance mGluR5 expression in the brain and spinal cord in ALS mouse model. In addition, mGluR5 expression increased in the whole brain significantly and moderately in the spinal cord during progression of the disease. Inflammation increased in all brain areas and spinal cord up to fivefold more than mGluR5 expression during progression of the disease indicating the excitatory role of excessive glutamate may contribute or accelerate of inflammation in ALS pathology.

## Abbreviations

[^11^C]PBR28: peripheral benzodiazepine receptor ligand 28
[^18^F]FPEB: [^18^F]3-fluoro-5-(2-pyridylethynyl)benzonitrile
3D-MLEM: three-dimensional-minimum likelihood estimation method
ALS: amyotrophic lateral sclerosis
BP_ND_: binding potential using reference tissue model
CT: computerized tomography
DAB: 3,3′-diaminobenzidine
IBA1: marker for inflammation (calcium binding adaptor molecule)
IHS: immunohistological studies
mCi: milli Curie
mGluR5: metabotropic glutamate receptor subtype 5
PET: positron emission tomography
PFA: paraformaldehyde
SOD1-G93A: superoxide dismutase 1-glycine 93S mutation (glycine 93 changed to alanine)
TSPO: translocator protein 18 kDA (kilo Dalton)
μmol: micromole

## References

[1] Strong MJ (2003). The basic aspects of therapeutics in amyotrophic lateral sclerosis. Pharmacology and Therapeutics..

[2] Corcia P, Gordon PH (2012). Amyotrophic lateral sclerosis and the clinical potential of dexpramipexole. Ther Clin Risk Manag..

[3] Dibaj P, Zschuntzsch J, Steffens H, Scheffel J, Goricke B, Weishaupt JH (2012). Influence of methylene blue on microglia-induced inflammation and motor neuron degeneration in the SOD1(G93A) model for ALS. PLoS One.

[4] Evans MC, Couch Y, Sibson N, Turner MR (2013). Inflammation and neurovascular changes in amyotrophic lateral sclerosis. Mol Cell Neurosci..

[5] Turner MR, Cagnin A, Turkheimer FE, Miller CC, Shaw CE, Brooks DJ (2004). Evidence of widespread cerebral microglial activation in amyotrophic lateral sclerosis: an [11C](R)-PK11195 positron emission tomography study. Neurobiol Dis.

[6] Giribaldi F, Milanese M, Bonifacio T, Anna Rossi PI, Di Prisco S, Pittaluga A (2013). Group I metabotropic glutamate autoreceptors induce abnormal glutamate exocytosis in a mouse model of amyotrophic lateral sclerosis. Neuropharmacology..

[7] Kanki R, Nakamizo T, Yamashita H, Kihara T, Sawada H, Uemura K (2004). Effects of mitochondrial dysfunction on glutamate receptor-mediated neurotoxicity in cultured rat spinal motor neurons. Brain Res.

[8] McDonnell M, Vera MD, Blass BE, Pelletier JC, King RC, Fernandez-Metzler C (2012). Riluzole prodrugs for melanoma and ALS: design, synthesis, and in vitro metabolic profiling. Bioorg Med Chem.

[9] Rahn KA, Slusher BS, Kaplin AI (2012). Glutamate in CNS neurodegeneration and cognition and its regulation by GCPII inhibition. Curr Med Chem.

[10] Spalloni A, Nutini M, Longone P (2013). Role of the N-methyl-d-aspartate receptors complex in amyotrophic lateral sclerosis. Biochim Biophys Acta.

[11] Yin HZ, Nalbandian A, Hsu CI, Li S, Llewellyn KJ, Mozaffar T (2012). Slow development of ALS-like spinal cord pathology in mutant valosin-containing protein gene knock-in mice. Cell Death Dis..

[12] Ghiasi P, Hosseinkhani S, Noori A, Nafissi S, Khajeh K (2012). Mitochondrial complex I deficiency and ATP/ADP ratio in lymphocytes of amyotrophic lateral sclerosis patients. Neurol Res.

[13] Shaw CE, al-Chalabi A, Leigh N (2001). Progress in the pathogenesis of amyotrophic lateral sclerosis. Curr Neurol Neurosci Rep.

[14] Turner BJ, Atkin JD, Farg MA, da Zang W, Rembach A, Lopes EC (2005). Impaired extracellular secretion of mutant superoxide dismutase 1 associates with neurotoxicity in familial amyotrophic lateral sclerosis. J Neurosci.

[15] Turner MR, Hardiman O, Benatar M, Brooks BR, Chio A, de Carvalho M (2013). Controversies and priorities in amyotrophic lateral sclerosis. Lancet Neurol.

[16] Miller T, Cleveland DW (2005). Treating neurodegenerative diseases with antibiotics. Science..

[17] Rothstein J, Patel S, Regan MR, Haenggeli C, Huang YH, Bergles DE (2005). Beta-lactam antibiotics offer neuroprotection by increasing glutamate transporter expression. Nature..

[18] Niswender C, Jones CK, Conn PJ (2005). New therapeutic frontiers for metabotropic glutamate receptors. Curr Top Med Chem..

[19] Pin P, Duvoisin R (1995). The metabotropic glutamate receptors: structure and functions. Neuropharmacol.

[20] Annoura H, Fukunaga A, Uesugi M, Tatsuoka T, Horikawa Y (1996). A novel class of antagonists for metabotropic glutamate receptors, 7-(hydroxyimino)cyclopropachromen-1a-carboxylates. Bioorg Med Chem Lett..

[21] Ritzen A, Mathiesen JM, Thomsen C (2005). Molecular pharmacology and therapeutic prospects of metabotropic glutamate receptor allosteric modulators. Basic Clin Pharmacol Toxicol.

[22] Zhang Z, Brownell A-L. Imaging of metabotropic glutamate receptors. In: Bright P, Ruskin A, editors. Neuroimaging- Clinical Applications. Rijeka, Croatia: InTech-Open Access Publisher; 2012. p. 499–532

[23] Williams JD, Lindsley CW (2005). Discovery of positive allosteric modulators of metabotropic glutamate receptor subtype 5 (mGluR5). Curr Top Med Chem..

[24] Aronica E, Catania MV, Geurts J, Yankaya B, Troost D (2001). Immunohistochemical localization of group I and II metabotropic glutamate receptors in control and amyotrophic lateral sclerosis human spinal cord: upregulation in reactive astrocytes. Neuroscience.

[25] O'Callaghan JP, Sriram K, Miller DB (2008). Defining "neuroinflammation". Ann N Y Acad Sci.

[26] Appel S, Zhao W, Beers DR, Henkel JS (2011). The microglial-motoneuron dialogue in ALS. Acta Myol.

[27] Wang J, Tueckmantel W, Zhu A, Pellegrino D, Brownell A-L (2007). Synthesis and preliminary biological evaluation of 3-[(18)F]fluoro-5-(2-pyridinylethynyl)benzonitrile as a PET radiotracer for imaging metabotropic glutamate receptor subtype 5. Synapse.

[28] Yu M, Tueckmantel W, Wang X, Zhu A, Kozikowski AP, Brownell A-L (2005). Methoxyphenylethynyl, methoxypyridylethynyl and phenylethynyl derivatives of pyridine: synthesis, radiolabeling and evaluation of new PET ligands for metabotropic glutamate subtype 5 receptors. Nucl Med Biol.

[29] Zhu A, Wang X, Yu M, Wang JQ, Brownell A-L (2007). Evaluation of four pyridine analogs to characterize 6-OHDA-induced modulation of mGluR5 function in rat brain using microPET studies. J Cereb Blood Flow Metab.

[30] Pellegrino D, Cicchetti F, Wang X, Zhu A, Yu M, Saint-Pierre M (2007). Modulation of brain dopaminergic and glutamatergic functions: microPET imaging studies in Parkinsonian rats. J Nucl Med.

[31] Sanchez-Pernaute R, Wang JQ, Kuruppu D, Cao L, Tueckmantel W, Kozikowski A (2008). Enhanced binding of metabotropic glutamate receptor type 5 (mGluR5) PET tracers in the brain of parkinsonian primates. Neuroimage.

[32] Wang JQ, Brownell A-L (2007). Development of metabotropic glutamate receptor ligands for neuroimaging. Curr Med Imag Rev..

[33] Logan J, Wolf AP, Shiue CY, Fowler JS (1987). Kinetic modeling of receptor-ligand binding applied to positron emission tomographic studies with neuroleptic tracers. J Neurochem..

[34] Logan J, Fowler JS, Volkow ND, Wolf AP, Dewey SL, Schlyer DJ (1990). Graphical analysis of reversible radioligand binding from time-activity measurements applied to [N-11C-methyl]- (−)-cocaine PET studies in human subjects. J Cereb Blood Flow Metab.

[35] Knippenberg S, Thau N, Dengler R, Petri S (2010). Significance of behavioral tests in a transgenic mouse model of amyotrophic lateral sclerosis. Behav brain Res.

[36] Fujita M, Imaizumi M, Zoghbi SS, Fujimura Y, Farris AG, Suhara T (2008). Kinetic analysis in healthy humans of a novel positron emission tomography radioligand to image the peripheral benzodiazepine receptor, a potential biomarker for inflammation. Neuroimage.

[37] Kreisl WC, Jenko KJ, HinesC S, Lyoo CH, Corona W, Morse CL (2013). A genetic polymorphism for translocator protein 18 kDa affects both in vitro and in vivo radioligand binding in human brain to this putative biomarker of neuroinflammation. J Cereb Blood Flow Metab.

[38] Zürcher NR, Loggia ML, Lawson R, Chonde DB, Izquierdo-Garcia D, Yasek JE (2015). Increased in vivo glial activation in patients with amyotrophic lateral sclerosis: assessed with [(11)C]-PBR28. Neuroimage Clin..

